# L-Ascorbate Attenuates the Endotoxin-Induced Production of Inflammatory Mediators by Inhibiting MAPK Activation and NF-κB Translocation in Cortical Neurons/Glia Cocultures

**DOI:** 10.1371/journal.pone.0097276

**Published:** 2014-07-01

**Authors:** Ya-Ni Huang, Chien-Cheng Lai, Chien-Tsai Chiu, Jhen-Jhe Lin, Jia-Yi Wang

**Affiliations:** 1 Department of Nursing, Hsin Sheng Junior College of Medical Care and Management, Taoyuan, Taiwan; 2 Division of Orthopedics, Department of Surgery, Far Eastern Memorial Hospital, New Taipei City, Taiwan; 3 Department of Neurosurgery, En Chu Kong Hospital, New Taipei City, Taiwan; 4 Graduate Institute of Medical Sciences and Department of Physiology, College of Medicine, Taipei Medical University, Taipei, Taiwan; French National Centre for Scientific Research, France

## Abstract

In response to acute insults to the central nervous system, such as pathogen invasion or neuronal injuries, glial cells become activated and secrete inflammatory mediators such as nitric oxide (NO), cytokines, and chemokines. This neuroinflammation plays a crucial role in the pathophysiology of chronic neurodegenerative diseases. Endogenous ascorbate levels are significantly decreased among patients with septic encephalopathy. Using the bacterial endotoxin lipopolysaccharide (LPS) to induce neuroinflammation in primary neuron/glia cocultures, we investigated how L-ascorbate (vitamin C; Vit. C) affected neuroinflammation. LPS (100 ng/ml) induced the expression of inducible NO synthase (iNOS) and the production of NO, interleukin (IL)-6, and macrophage inflammatory protein-2 (MIP-2/CXCL2) in a time-dependent manner; however, cotreatment with Vit. C (5 or 10 mM) attenuated the LPS-induced iNOS expression and production of NO, IL-6, and MIP-2 production. The morphological features revealed after immunocytochemical staining confirmed that Vit. C suppressed LPS-induced astrocytic and microglial activation. Because Vit. C can be transported into neurons and glia via the sodium-dependent Vit. C transporter-2, we examined how Vit. C affected LPS-activated intracellular signaling in neuron/glia cocultures. The results indicated the increased activation (caused by phosphorylation) of mitogen-activated protein kinases (MAPKs), such as p38 at 30 min and extracellular signal-regulated kinases (ERKs) at 180 min after LPS treatment. The inhibition of p38 and ERK MAPK suppressed the LPS-induced production of inflammatory mediators. Vit. C also inhibited the LPS-induced activation of p38 and ERK. Combined treatments of Vit. C and the inhibitors of p38 and ERK yielded no additional inhibition compared with using the inhibitors alone, suggesting that Vit. C functions through the same signaling pathway (i.e., MAPK) as these inhibitors. Vit. C also reduced LPS-induced IκB-α degradation and NF-κB translocation. Thus, Vit. C suppressed the LPS-stimulated production of inflammatory mediators in neuron/glia cocultures by inhibiting the MAPK and NF-κB signaling pathways.

## Introduction

Neuroinflammation plays a crucial role in the pathogenesis of not only acute brain insults, such as bacterial infections [1], [2], cerebral ischemia [3], and traumatic brain injury [4], but also in chronic neurodegenerative diseases such as Alzheimer's disease [5]. Neuroinflammation involves a complex interplay of glia cells beginning in microglial cells, which activate astrocytes (reactive gliosis) resulting in the release of inflammatory molecules [4], [6] that can cause neuronal damage. In an animal model, glial cells, and particularly astrocytes and microglia, have been shown to provide the early sources of proinflammatory cytokines in cerebrospinal fluid (CSF) and brain tissues when meningoencephalitis is caused by a Gram-negative bacterium (*Klebsiella* pneumonia) infection [1], [2]. Cell culture studies have demonstrated that glial cells activated by lipopolysaccharide (LPS), a Gram-negative bacterial cell wall endotoxin, time-dependently expressed inducible nitric oxide synthase (iNOS) [7] and proinflammatory cytokines and chemokines [8]. Toll-like receptor 4 (TLR4) is the receptor of LPS and is expressed in neurons and glial cells [9]. The activation of TLR4 by LPS causes the activation of mitogen-activated protein kinases (MAPKs), including extracellular signal-regulated kinase (ERK), p38, c-Jun N-terminal kinase (JNK), and the nuclear factor (NF)-κB signaling pathway in brain cells, yielding neuroinflammation [9], [10].

It has been demonstrated that cultured astrocytes treated with LPS plus interferon (IFN)-γ deplete endogenous antioxidant Vit. C, causing increased iNOS expression [11]. Ischemia [12] and bacterial meningitis [13] can cause the loss of Vit. C in the brain, implying that this loss is pathophysiologically relevant. The highest concentrations of Vit. C in the body are observed in brain cells [14], [15], and Vit. C is a vital antioxidant molecule in the brain. The physiological concentration of Vit. C occurs in the millimolar range in neurons (10 mM) and glia (1 mM) [16]. Various crucial functions of Vit. C are involved in cellular reactions. In addition to its role as an antioxidant, Vit. C serves as a cofactor in several enzyme reactions, including those involved in the biosynthesis of collagen, carnitine, and norepinephrine [16], [17]. Vit. C can also inhibit the TNF-α-induced activation of NF-κB through the activation of p38 MAPK in endothelial cells [18]. This suggests that Vit. C is a regulator of cytokine redox-signal transduction in host defense cells and plays a role in controlling inflammatory responses [19].

Vit. C must be exogenously administered because of the lack of the terminal enzyme, L-gulonolactone oxidase, the last enzyme in the pathway for synthesizing ascorbic acid from glucose [16], [20] in humans, primates, and guinea pigs. Thus, Vit. C must be absorbed from exogenous sources, transported to the brain, and taken into cells via a Vit. C transporter [15]. Pretreatment with Vit. C attenuates the induction of iNOS expression and inhibition of glutamate uptake after treating cultured astrocytes with LPS and IFN-γ [11]. In addition, it has been demonstrated that Vit. C is beneficial in animal models of stroke [21] and Alzheimer's disease [22]. These results suggest that Vit. C treatment can ameliorate neuroinflammation; however, the underlying mechanisms by which Vit. C affects neuroinflammation remain unclear.

LPS initiates a neuroinflammatory cascade characterized by the activation of glia cells and increased production of inflammatory mediators. Hence, controlling activated glia at the levels of the TLR4 or signaling pathways, including the phosphorylation of MAPKs (such as p38 and ERK) and NF-κB translocation, may provide a therapeutic strategy for addressing neuroinflammation. The excessive production of inflammatory mediators can be a functional index of activated glia. Using neuron/glia cocultures, we investigated how Vit. C affected LPS-induced neuroinflammatory responses, exploring whether the effects of Vit. C were mediated through the MAPK pathways or NF-κB translocation.

## Materials and Methods

### Chemical reagents and antibodies

Sodium-L-ascorbate (Vit. C) and LPS were purchased from Sigma-Aldrich (St. Louis, MO, USA). The p38 MAPK inhibitor, SB203580; ERK inhibitor, PD98059; iNOS; and β-actin were purchased from Calbiochem (San Diego, CA, USA). Antibodies against p38, phospho-p38 (p-p38), ERK, phospho-ERK (p-ERK), and IκB-α were purchased from Cell Signaling Technology (Beverly, MA, USA). Antibodies against p65 and poly (ADP-ribose) polymerase (PARP) were purchased from Cell Signaling Technology and Santa Cruz Biotechnology (CA, USA), respectively.

### Primary rat cortical neuron/glia cocultures

Neuron/glia cocultures were prepared from the cerebral cortices of 1-d-old neonatal Sprague-Dawley rats. These procedures were performed according to the National Institutes of Health Guidelines for the Care and Use of Laboratory Animals and were approved by the Institutional Animal Care and Use Committee of Taipei Medical University (Taipei, Taiwan). After the rats were sacrificed, their brains were quickly removed. The dissected cerebral cortices were placed in ice-cold Hank's solution (without Ca^2+^ or Mg^2+^) [23]. Subsequently, cortical cells were dissociated by trituration using a pipette. Following centrifugation (1500 rpm for 5 min), the cells were suspended in 10% fetal bovine serum and Dulbecco's modified Eagle's medium (Gibco BRL, Grand Island, NY, USA), seeded at a density of 5 × 10^5^ cells/mL, and incubated at 37 °C in humidified 5% CO_2_/95% air. The percentage cell composition was determined using immunostaining, followed by cell counting. The neuron/glia cocultures consisted of approximately 40%–42% neurons, 40%–45% astrocytes, and 8%–10% microglia. Other unstained cell types, such as oligodendrocytes, fibroblasts, and smooth muscle cells, represented <2% of the coculture.

### Measuring nitrite accumulation

Nitrite, a downstream product of NO, was measured using the Griess reaction in culture medium. Briefly, culture medium was mixed with an equal volume of Griess reagent. The absorbance was determined at 540 nm by using a microplate reader (Molecular Devices, Menlo Park, CA, USA).

### Measuring IL-6 and MIP-2

The concentrations of IL-6 and MIP-2 secreted into the culture medium were measured using enzyme-linked immunosorbent assay (ELISA) kits (BioSource International, Camarillo, CA, USA) according to the manufacturer protocol.

### Extracting cytoplasmic and nuclear proteins

At 1 h after treatment, the cultured cells were harvested and washed three times with cold phosphate-buffered saline (PBS). The cytoplasmic and nuclear protein fractions were extracted using NE-PER extraction reagent (Thermo Fisher Scientific, Rockford, IL, USA) according to the manufacturer protocol and used for Western blotting.

### Western blotting

After employing the relevant treatments, the cells were harvested and lysed in protein extraction buffer (Mammalian Cell-PE LB, Geno Technology, USA) containing protease and phosphatase inhibitors (Complete Mini, Roche Diagnostics, Indianapolis, IN, USA). The lysed cells were centrifuged at 12000 rpm for 10 min (at 4 °C), and the supernatant was collected for storage at −80 °C or for use in the Western blot analysis. The proteins were separated using gel electrophoresis and electroblotted onto polyvinylidene difluoride membranes (PerkinElmer Life Sciences, USA). The membranes were blocked with 5% nonfat milk and incubated overnight at 4 °C with the indicated antibodies, namely iNOS (1∶1000 dilution), p38, p-p38, ERK, p-ERK and IκB-α and p65 (1∶1000 dilution), followed by blocking with an appropriate secondary antibody (1∶20000 dilution) for 1 h at room temperature (RT). Signals were visualized using enhanced chemiluminescent detection reagents (PerkinElmer Life Sciences). The membrane was then stripped and reprobed using an antibody specific to β-actin (cytoplasmic loading control) or PARP (nuclear loading control) (1∶10,000 dilution) to ensure the accuracy of each loading. The level of protein expression was quantified using a BioImaging System (Level Biotechnology).

### 3-(4,5-Dimethylthianol-2-yl)-2,5 diphenyl tetrazolium bromide (MTT) reduction assay

A colorimetric MTT reduction assay was performed to measure cell as previously described [24]. Each culture well was incubated in 0.5 mg/ml of MTT culture medium, followed by a 40-min incubation in 5% CO_2_ at RT. The culture medium was then aspirated, and the cells were lysed with 50% dimethyl sulfoxide. The MTT reduction was quantified by measuring the absorbance at a test wavelength of 570 nm and a reference wavelength of 630 nm (the 630-nm reading was subtracted from the 570-nm reading) by using a microplate reader (Molecular Devices, Menlo Park, CA).

### Measuring the release of lactate dehydrogenase (LDH)

The level of cytotoxicity was assayed based on the level of LDH activity in the culture media and scaled to the value of maximal death ( =  100%) measured after a freeze-thaw treatment, as a positive control (Pos.). Briefly, the freeze-thaw was performed in 3 cycles of 60 min, freezing at −80 °C and thawing at 37 °C, after the initial period of freezing overnight. The level of cytotoxicity was presented as the percentage of values relative to the Pos. cultures.

### Immunocytochemical staining

After the experiment, the cultures were fixed in 4% paraformaldehyde and permeated using 0.2% Triton X-100 in PBS for 10 min, as previously described [23], [25]. The background staining was reduced by blocking nonspecific binding sites with 10% goat serum for 1 h at RT. After rinsing with PBS, the endogenous peroxidase activity was quenched by incubation with 3% H_2_O_2_ in PBS for 10 min. The cultures were rinsed again and incubated overnight with appropriate primary antibodies (mouse anti-NeuN, 1:500, Chemicon, Temecula, CA, USA; mouse anti-GFAP, 1:1000, Chemicon; and mouse anti-ED1, 1:500, Serotec, Bicester, UK) at 4 °C. The cells were then rinsed three times with PBS and incubated with a secondary antibody (biotinylated antimouse immunoglobulin G, at 1:200, Vector Laboratories Burlingame, CA, USA) for 1 h. After incubation, the cells were washed with PBS three times (15 min per wash) and visualized using the avidin-biotin peroxidase complex method (ABC Elite kit; Vector Laboratories).

### Statistical analysis

All data are presented as the mean ± the standard error of the mean (SEM). The level of statistical significance was assessed using a one-way analysis of variance followed by the Student-Newman-Keuls' test, using the SigmaStat program (Jandel Scientific, San Rafael, CA, USA). A value of *p*<.05 represented a significant difference compared with the indicated experimental group.

## Results

### LPS-induced iNOS expression, NO production, and the release of proinflammatory mediators

**Figure 1:**
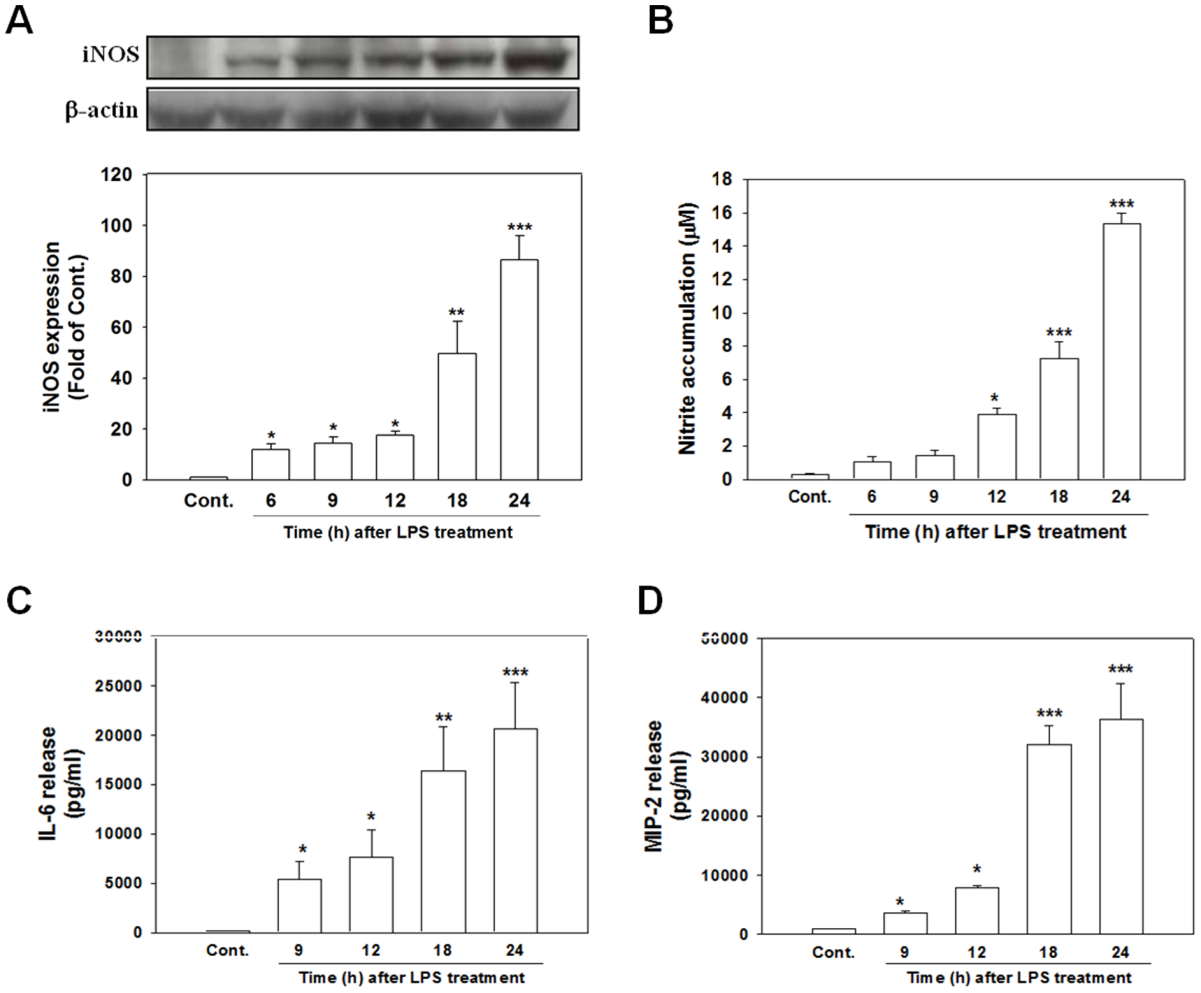
LPS stimulated the production of inflammatory mediators at various times. Cultured cells were treated with LPS (100 ng/ml) or PBS (control; Cont.) for the indicated time periods. The cells were harvested to determine the level of iNOS expression by using western blotting; (A) the culture medium was collected to analyze the levels of nitrite (B); IL-6 (C); and MIP-2 (D). The nitrite accumulation was measured using Griess reagent. IL-6 and MIP-2 production was measured using ELISA kits. The data are represented as mean ± SEM based on 4–6 independent experiments. * *p*<.05, ** *p*<.01, and *** *p*<.001 versus the control (Cont.).

To examine whether LPS elicits neuroinflammatory responses, such as inducing iNOS and the release of proinflammatory mediators, we first examined the time course of LPS-induced iNOS expression, nitrite accumulation, and the release of IL-6 (a cytokine) and MIP-2 (a chemokine) in neuron/glia cocultures. As shown in Fig. 1, treating cells with LPS (100 ng/ml) for various times (6, 9, 12, 18, and 24 h) induced the time-dependent expression of iNOS and accumulation of nitrite. The western blot results indicated that the expression of the iNOS protein was negligible in the control cultures; by contrast, significant levels of iNOS protein expression occurred as early as 6 h (*p*<.05) and gradually increased over time at 9 (*p*<.05), 12 (*p*<.05), 18 (*p*<.01), and 24 h (*p*<.001) after LPS treatment (Fig. 1A). NO production, as reflected by levels of nitrite accumulation, significantly increased in the cultured cells exposed to LPS for 12 h (*p*<.05) and continued to increase from 18 to 24 h (*p*<.001; Fig. 1B). We also found that IL-6 and MIP-2 significantly increased in the cultured cells exposed to LPS for 9 h and remained elevated at up to 24 h (*p*<.001; Figs. 1C and 1D). These results suggest that the LPS-induced activation of glial cells to elicit neuroinflammatory responses.

### Involvement of p38 and ERK MAPK signaling pathways in the LPS-induced production of inflammatory mediators

**Figure 2:**
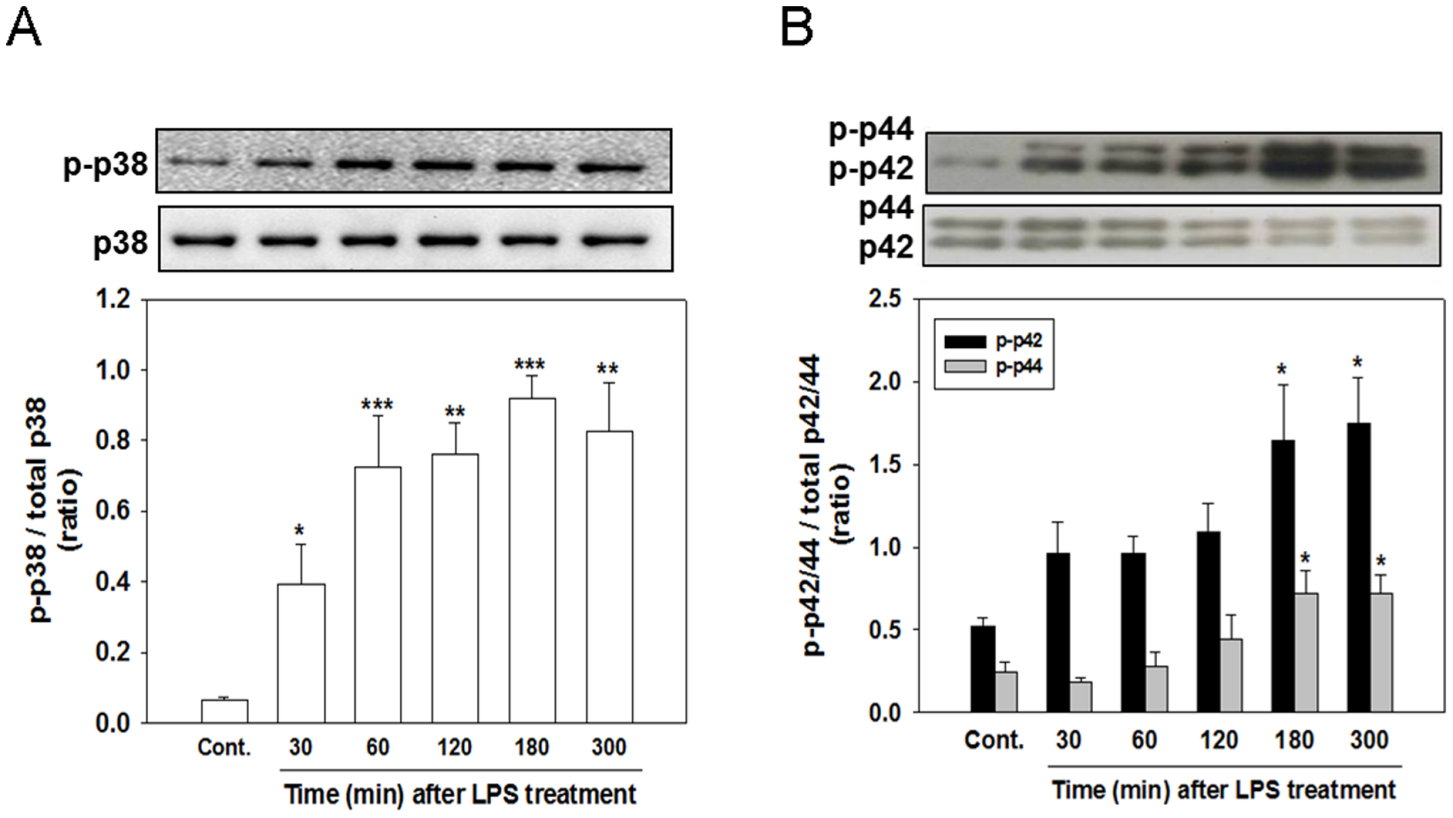
Time course of the LPS-stimulated phosphorylation of p38 (p-p38) and ERK (p-ERK). Cocultured cells were treated with LPS (100 ng/ml) for the indicated times (30, 60, 120, 180, and 300 min). The cells were harvested to analyze the levels of p-p38 (A) and p-ERK (p-p42/44) (B) protein expression by using western blotting. LPS stimulated the phosphorylation of both p38 and ERK, but the phosphorylation of p38 appeared at 60-min post LPS, whereas that of ERK appeared substantially later at 180 min. The data are represented as mean ± SEM based on 3–4 independent experiments. * *p*<.05, ** *p*<.01, and *** *p*<.001 versus Cont.

To examine whether the activation of p38 and ERK MAPKs are involved in LPS-induced neuroinflammatory responses, we examined the phosphorylation of p38 and ERK and the effects of inhibitors of p38 and ERK. Figure 2A shows that the level of phosphorylated p38 MAPK (p-p38) was significantly increased at 30 min after LPS treatment (*p<*.05); this level was maintained at 180 min (*p<*.001), but significantly declined at 300 min (*p<*.01). Figure 2B shows similar results, indicating a gradual increase in the levels of phosphorylated ERK (p-p42/44) at 180 min (*p<*.05 for p-p42 and, *p<*.05 for p-p44) and up to 300 min (*p<*.05).

**Figure 3:**
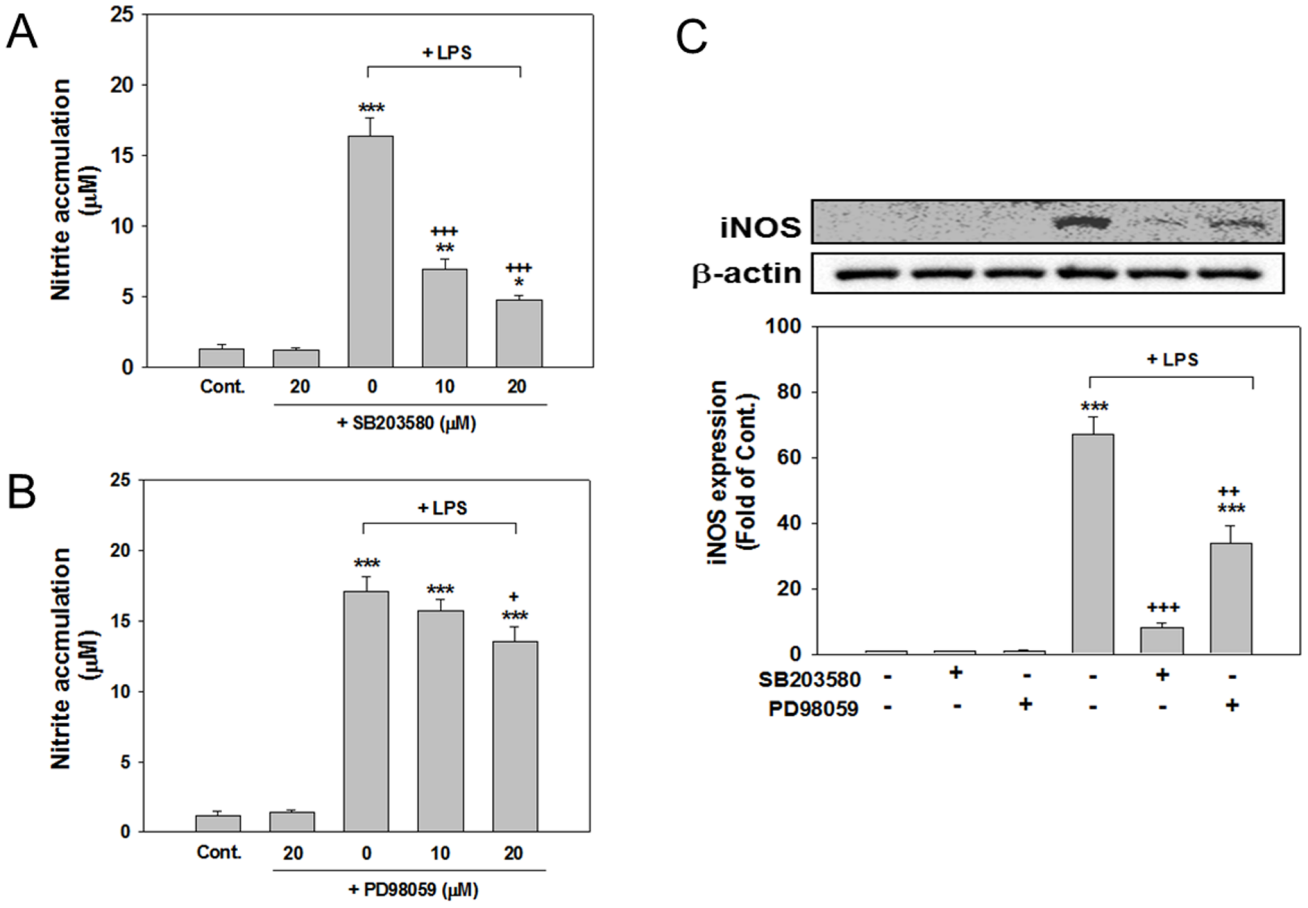
Effects of MAPK inhibitors on the levels of LPS-induced nitrite accumulation and iNOS expression. Cocultured cells were treated for 24 µM of SB203580 (a p38 MAPK inhibitor) or PD98059 (an ERK MAPK inhibitor), which were simultaneously with LPS (100 ng/ml). SB203580 (A) and PD98059 (B) significantly suppressed the LPS-induced accumulation of nitrite and expression of iNOS (C). The data are represented as mean ± SEM based on 3–5 independent experiments. * *p*<.05, ** *p*<.01, and *** *p*<.001 versus Cont.; ^+^ *p*<.05,^ ++^ *p*<.01 and ^+++^ *p*<.001 versus LPS alone.

To further determine whether MAPKs (p38 and ERK) are involved in the production of LPS-induced inflammatory mediators, we treated cells with 10 or 20 µM of SB203580 (a p38 MAPK inhibitor) or PD98059 (an ERK MAPK inhibitor) and observed how these inhibitors affected the levels of nitrite accumulation, iNOS expression, IL-6 and MIP-2 release. According to the results, in the absence of LPS, neither SB203580 nor PD98059 at 10 or 20 µM significantly affected the nitrite accumulation compared with the control cultures (because the results were similar at 10 and 20 µM, only data for 20 µM are shown in Figs. 3A and 3B, second columns). By contrast, cotreating SB203580 (10 or 20 µM) with LPS for 24 h significantly inhibited the LPS-induced nitrite accumulation (*p*<.001), and 20 µM inhibited this accumulation more compared with 10 µM; however, the difference was nonsignificant. Cotreating 10 µM of PD98059 with LPS did not significantly reduce the nitrite accumulation, whereas 20 µM of cotreatment attained a significant reduction (*p*<.05). The concentration of 20 µM was adopted for both SB203580 and PD98059 in subsequent experiments. Regarding iNOS induction, treatment with 20 µM of inhibitors (SB203580 or PD98059) alone exerted no effects (Fig. 3C); however, western blot analyses indicated that cotreating these inhibitors for 24 h with LPS significantly reduced the level of LPS-induced iNOS expression. (*p<*.001 and *p<*.01, Fig. 3C).

**Figure 4:**
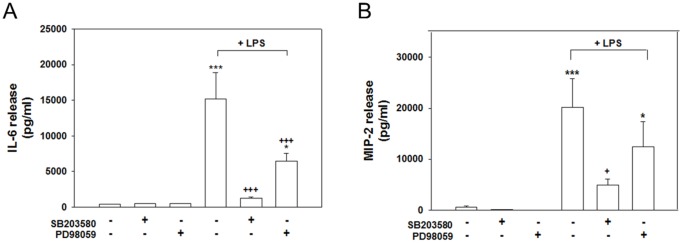
Effects of MAPK inhibitors on LPS-induced IL-6 and MIP-2 production. Cocultured cells were treated with 20 µM of the p38 inhibitor SB203580 or the ERK inhibitor PD98059, which were simultaneously added with LPS (100 ng/ml) for 24 h. The levels of IL-6 (A) and MIP-2 (B) in the medium were measured using ELISA kits. Both inhibitors significantly suppressed both the LPS stimulated release of IL-6 and MIP-2. The data are represented as mean ± SEM based on 4–6 independent experiments. * *p*<.05 and *** *p*<.001 versus Cont.; ^+^ *p*<.05 and ^+++^ *p*<.001 versus LPS alone.

In addition, cotreating cells with 20 µM SB203580 and LPS for 24 h significantly attenuated the release of IL-6 (*p*<.001) and MIP-2 (*p*<.05) compared with the release in the cultures treated with LPS alone (Figs. 4A and B). Cultured cells cotreated with 20 µM PD98059 and LPS for 24 h exhibited significantly reduced levels of IL-6 release (*p*<.001; Fig. 4A) but not MIP-2 release (Fig. 4B). The level of IL-6 and MIP-2 produced in cultures treated with SB203580 or PD98059 (20 µM; Fig. 4A) alone did not significantly differ from that produced in the control cultures (Fig. 4B). These results suggest that the activation of p38 and ERK MAPK is involved in the production of LPS-induced inflammatory mediators.

### Vit. C suppressed LPS-induced iNOS expression, nitrite accumulation, and the release of inflammatory mediators without causing cell death

**Figure 5:**
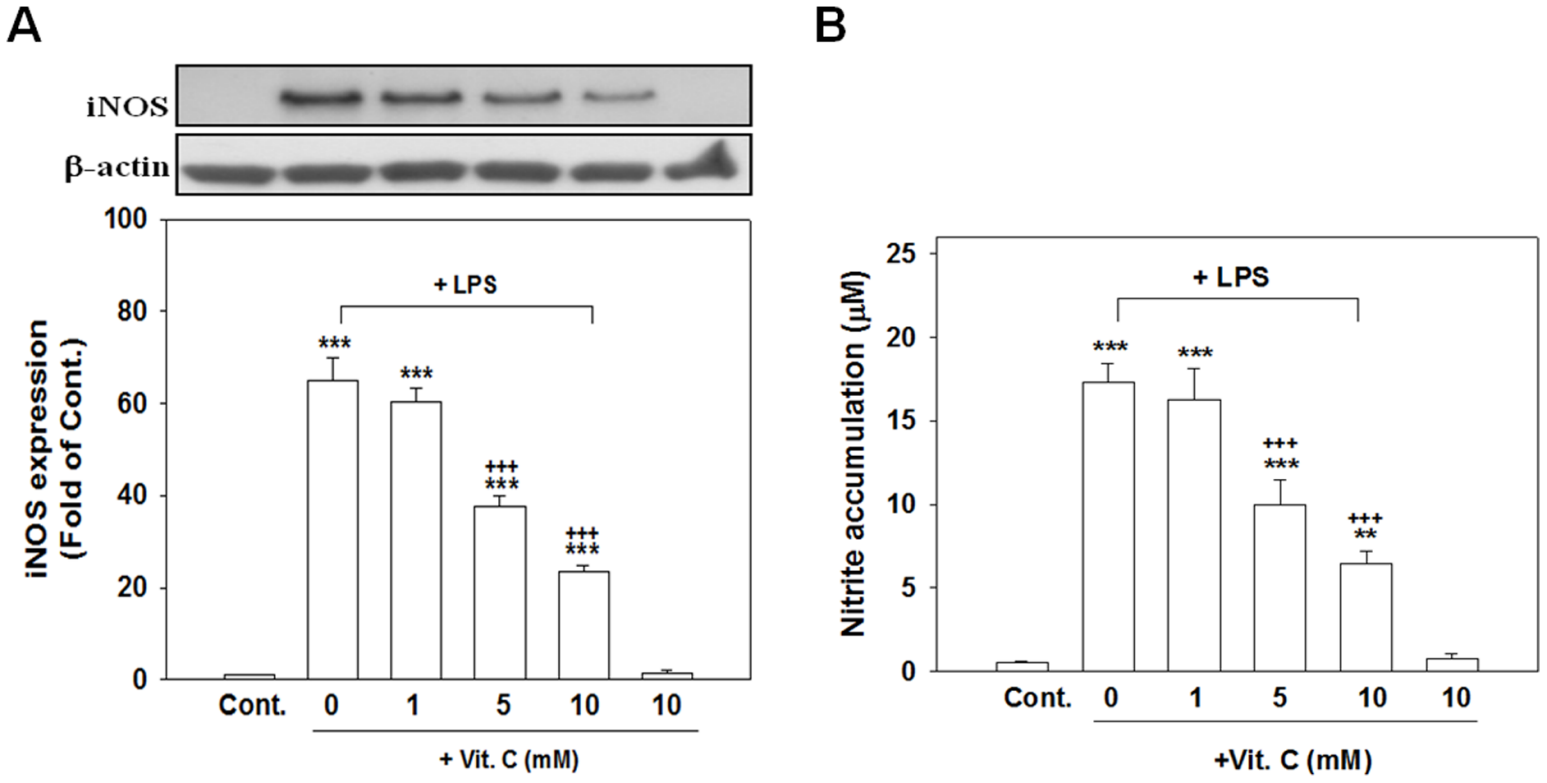
Vitamin C suppressed the LPS-induced expression of iNOS and nitrite accumulation. Cocultured cells were treated for 24(1, 5, and 10 mM), which were simultaneously added with LPS (100 ng/ml) and then harvested to conduct western blot analyses of iNOS expression (A). The level of nitrite accumulation (B) in the culture medium was measured using Griess reagent. Vit. C suppressed both the LPS-induced iNOS expression and nitrite accumulation. The data are represented as the mean ± SEM based on 4–6 independent experiments. ** *p*<.01 and *** *p*<.001 versus Cont.; ^+++^ *p*<.001 versus LPS alone

**Figure 6:**
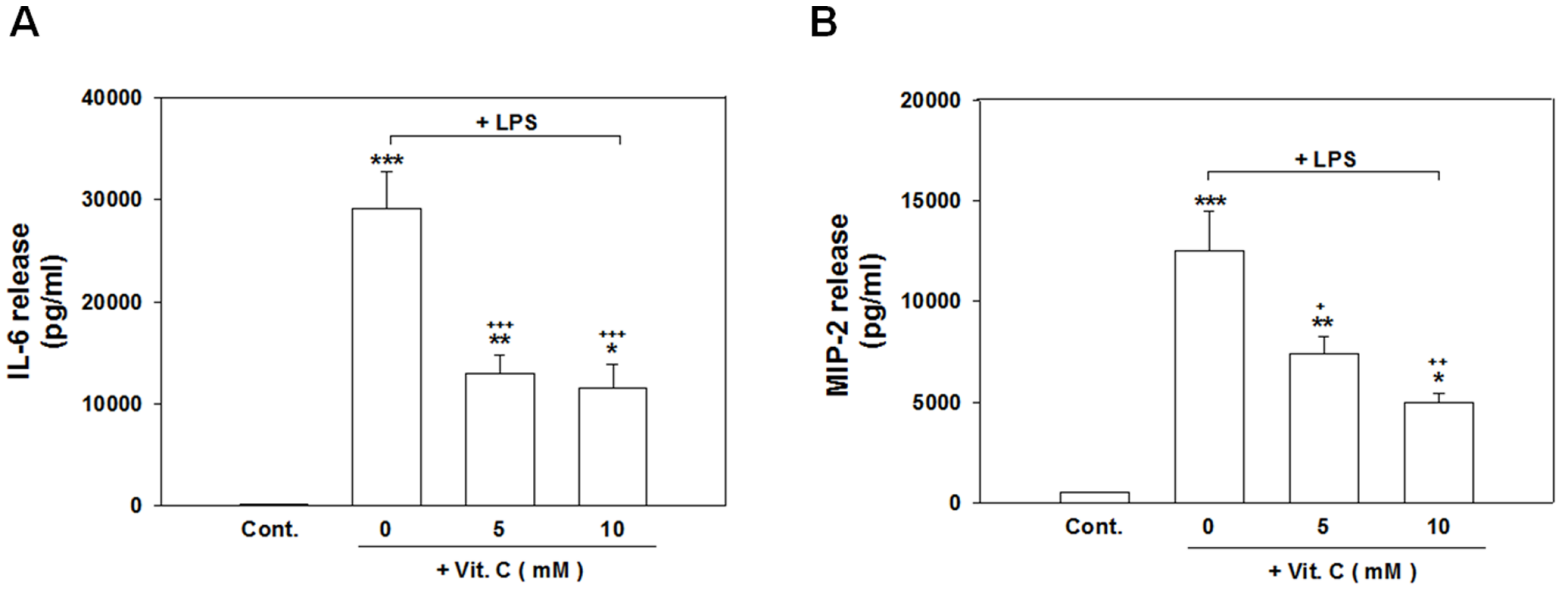
Vitamin C suppressed the LPS-induced production of IL-6 and MIP-2. Cocultured cells were treated for 24(100 ng/ml). The culture medium was collected and the levels of IL-6 (A) and MIP-2 (B) production were measured using ELISA kits. The data are represented as the mean ± SEM based on 4–6 independent experiments. * *p*<.05, ** *p*<.01, and *** *p*<.001 versus Cont.; ^+^ *p*<.05, ^++^ *p*<.01, and ^+++^ *p*<.001 versus LPS alone.

Vit. C typically serves as an endogenous regulator of cytokine redox-signal transduction in host defense cells. We examined whether exogenous Vit. C could attenuate an experimental inflammatory response produced using LPS in a neuronal/glial coculture model. Exposing cells to LPS for 24 h significantly enhanced the levels of iNOS expression (Fig. 5A; *p*<.001), nitrite accumulation (Fig. 5B; *p*<.001), and IL-6 (Fig 6A; *p*<.001) and MIP-2 (Fig 6B; *p*<.001) release compared with those of the control cultures. Cotreating cells with 5 and 10 mM but not 1 mM of Vit. C significantly reduced the level of LPS-induced iNOS protein expression (Fig. 5A; *p*<.001) and nitrite accumulation (Fig. 5B; *p*<.001). Vit. C (10 mM) alone exerted no effects (Figs. 5A and B).

Cotreatment with 5 or 10 mM of Vit. C and LPS for 24 h significantly attenuated the release of IL-6 (Fig. 6A; *p*<.001) and MIP-2 (Fig. 6B; *p*<.05 for 5 mM Vit. C and *p*<.01 for 10 mM Vit. C) compared with using LPS treatment alone.

**Figure 7:**
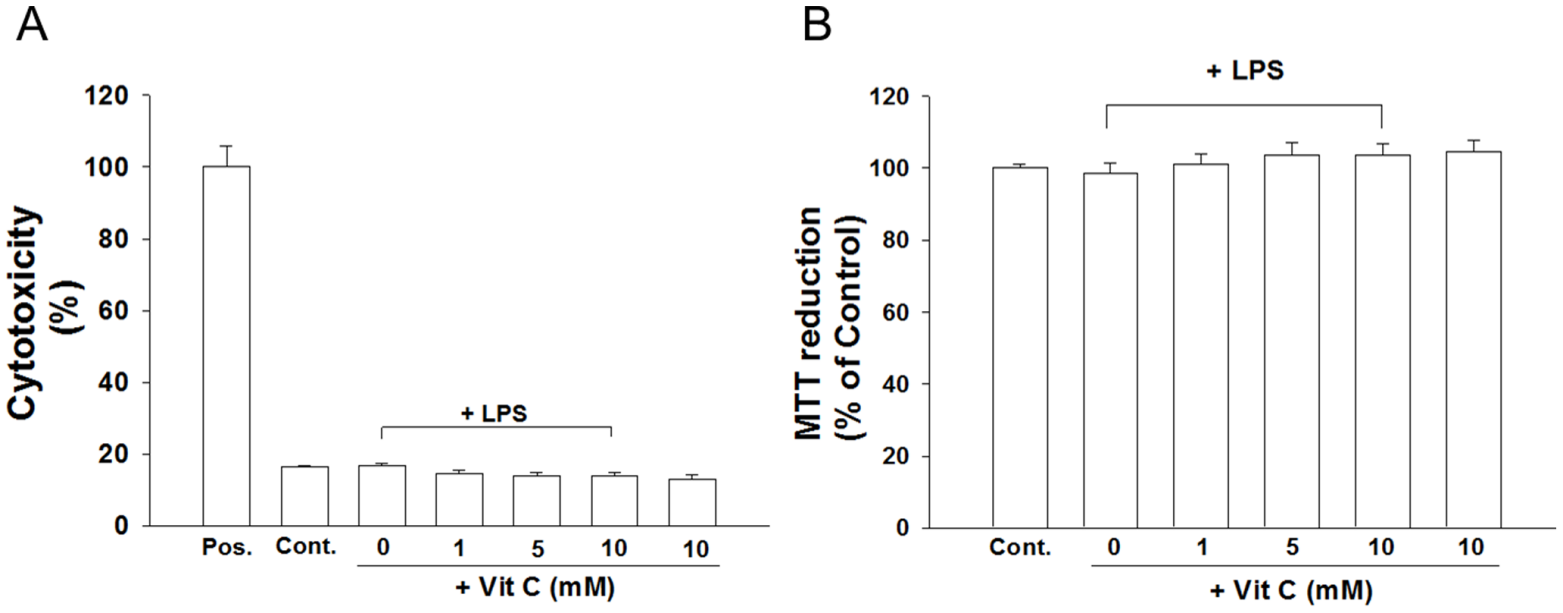
Lack of Vit. C (1, 5 10 mM) effects on cell survival (measured using an MTT reduction assay) and cell death (measured based on the level of LDH release). Cultured cells were treated for 24 h with various concentrations of Vit. C, which was simultaneously added with LPS (100 ng/ml). Selected concentrations of Vit. C (1, 5, 10 mM) did not affect the level of cell viability as assayed based on LDH release (A) and MTT reduction (B). The cytotoxicity was assayed based on the level of LDH activity in the culture media; this activity level was scaled to the value of maximal death ( = 100%) as a positive control (Pos.) after freeze-thaw treatment. The data are represented as the mean ± SEM based on 4–6 independent experiments.

We further examined whether the suppression of the LPS-induced release of inflammatory mediators by Vit. C was a result of the cytotoxicity of Vit. C. Cultured cells exposed to 10 mM Vit. C or LPS did not significantly differ from PBS-treated cells (Figs. 7A and B) regarding cell viability. Compared with the medium control, no significant differences existed in the levels of LDH release (an indicator of cell death) at various concentrations (1, 5, and 10 mM) when simultaneously adding Vit. C and LPS (Fig. 7A). Similarly, the MTT reduction assay showed that the simultaneous exposure to 1, 5, or 10 mM of Vit. C and LPS nonsignificantly affected the cell viability compared with that of the control cultures (Fig. 7B); thus, the selected concentrations of Vit. C were not toxic to cells. These results suggest that the selected Vit. C concentrations effectively ameliorated LPS-induced inflammation without damaging cells.

### Vitamin C attenuated the LPS-induced activation of astrocytes and microglia without affecting neurons as evidenced by immunocytochemical staining

**Figure 8:**
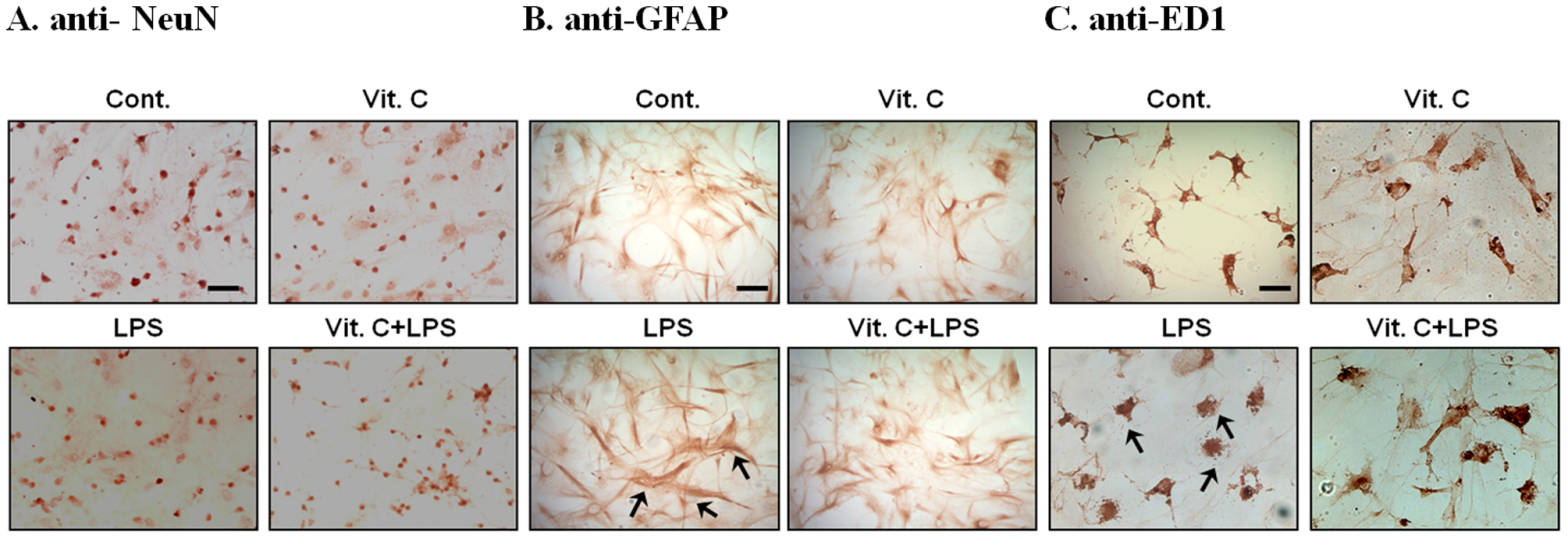
Morphological changes that various treatments exerted on cocultured neuronal/glial cells. Cultured cells were treated with PBS (Control), Vit. C, LPS, or a combination of Vit. C-LPS for 24 h, fixed, and then immunocytochemically stained with an antibody against a specific cell marker (NeuN, GFAP, or ED1). The representative photomicrographs show the cultures stained with antibodies to identify neurons (NeuN-positive cells; A), astrocytes (GFAP-positive cells; B), and microglia (ED1-positive cells; C). The arrows indicate the activated glial cells. Scale bar  =  50 µm.

Glial cells exhibit varied distinguishing morphological features in their resting and activated states, providing a convenient way to assess the activation status of such cells. To examine how Vit. C affected LPS-induced neuroinflammation, we immunocytochemically stained various cells with specific cell markers and evaluated any changes in their morphological features following treatment with LPS or Vit. C alone, or cotreatment with Vit. C and LPS. The results indicated that the neuron morphologies (NeuN-positive cells) did not significantly differ among the untreated control cells and the cells treated with Vit. C, LPS, or Vit. C-LPS cotreatment (Fig. 8A); however, LPS treatment changed the morphologies of both astrocytes (GFAP positive) and microglia (ED-1 positive) from those representing the resting state to those representing the active state. The activated astrocytes displayed enlarged (hypertrophic) size, numerous cytoplasmic processes, and a massive accumulation of GFAP-positive filaments (Fig. 8B); the ED-1 positive microglia changed from their resting forms, which exhibit a small soma and long cytoplasmic processes, to their active states, which are characterized by amoeboid appearances (Fig. 8C). Cotreatment with Vit. C and LPS attenuated the LPS-induced morphological changes in both the astrocytes (Fig. 8B) and microglia (Fig. 8C). Both astrocytes and microglia treated with Vit. C alone retained their resting morphologies. Although Vit. C alone exerted no effects the results suggested it could attenuate the activation of astrocytes and microglia when combined with LPS.

### Vitamin C inhibited the LPS-stimulated activation of p38 and ERK

**Figure 9:**
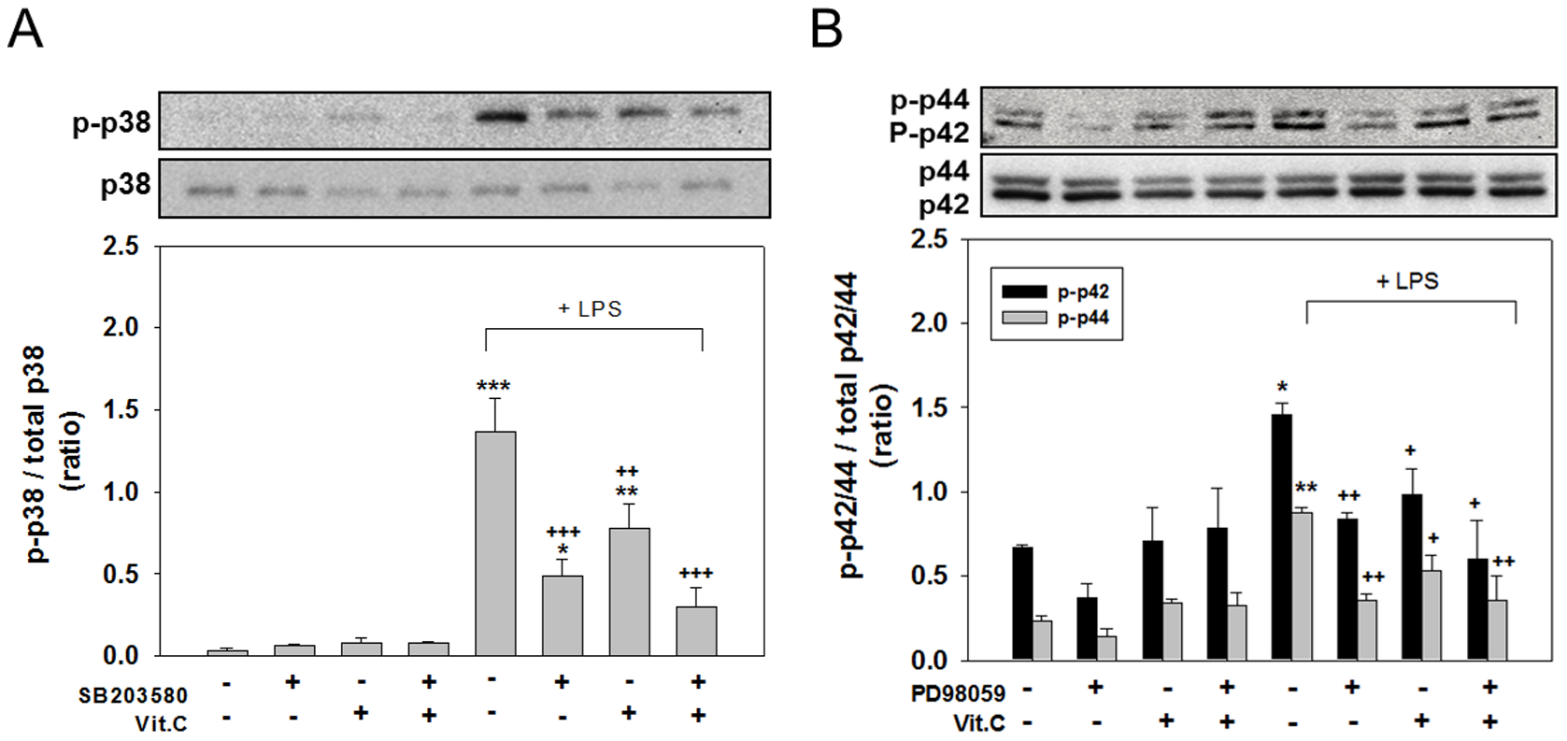
MAPK Inhibitors and Vit. C inhibited the LPS-induced phosphorylation of p38 and ERK. Cells were treated for 180(control), LPS (100 ng/ml) alone, or LPS combined with 20 µM of the p38 inhibitor SB203580 or ERK inhibitor PD98059, or 10 mM of Vit. C, and then harvested to conduct western blot analyses of p-p38 (A) and p-ERK (p-p42/44) (B). The data are represented as the mean ± SEM based on 3–4 independent experiments. ** *p*<.05, ** *p*<.01, and *** *p*<.001 versus Cont.; ^+^ *p*<.05, ^++^ *p*<.01 and ^+++^ *p*<.001 versus LPS alone.

To investigate whether the antiinflammatory effects of Vit. C were mediated by the inhibition of LPS-induced MAPK activation, we examined the effects of simultaneous treatments with LPS for 180 min by using various combinations of the p38-specific inhibitor SB203580, ERK inhibitor PD98058, and Vit. C. The western blot results indicated that the LPS-stimulated phosphorylation of p38 and p42/p44 was significantly inhibited by SB203580 (Fig. 9A; *p<*.001) and PD98059 (Fig. 9B; *p<*.05 for p-p42 and *p<*.01 for p-p44). Vit. C-LPS cotreatment significantly reduced the p-p38 (Fig. 9A; *p<*.01) and p-p42/44 levels (Fig. 9B; *p<*.05 for p-p42 and *p<*.05 for p-p44). Treatments combining Vit. C with SB203580 or PD98059 significantly reduced the LPS-induced p-p38 (Fig. 9A; *p<*.001) and p-p42/44 levels (Fig. 9B; *p<*.05 for p-p42 and *p<*.01 for p-p44). The activation of p38 and ERK (reflected by the p-p38 and p-p42/44 levels) did not significantly differ among the control cultures or those treated with inhibitors, Vit. C alone, or a combination of inhibitors and Vit. C. These results suggest that Vit. C suppressed the production of LPS-induced inflammatory mediators by inhibiting the p38 and ERK MAPK pathways.

### Vitamin C inhibited the LPS-induced translocation of NF-κB

**Figure 10:**
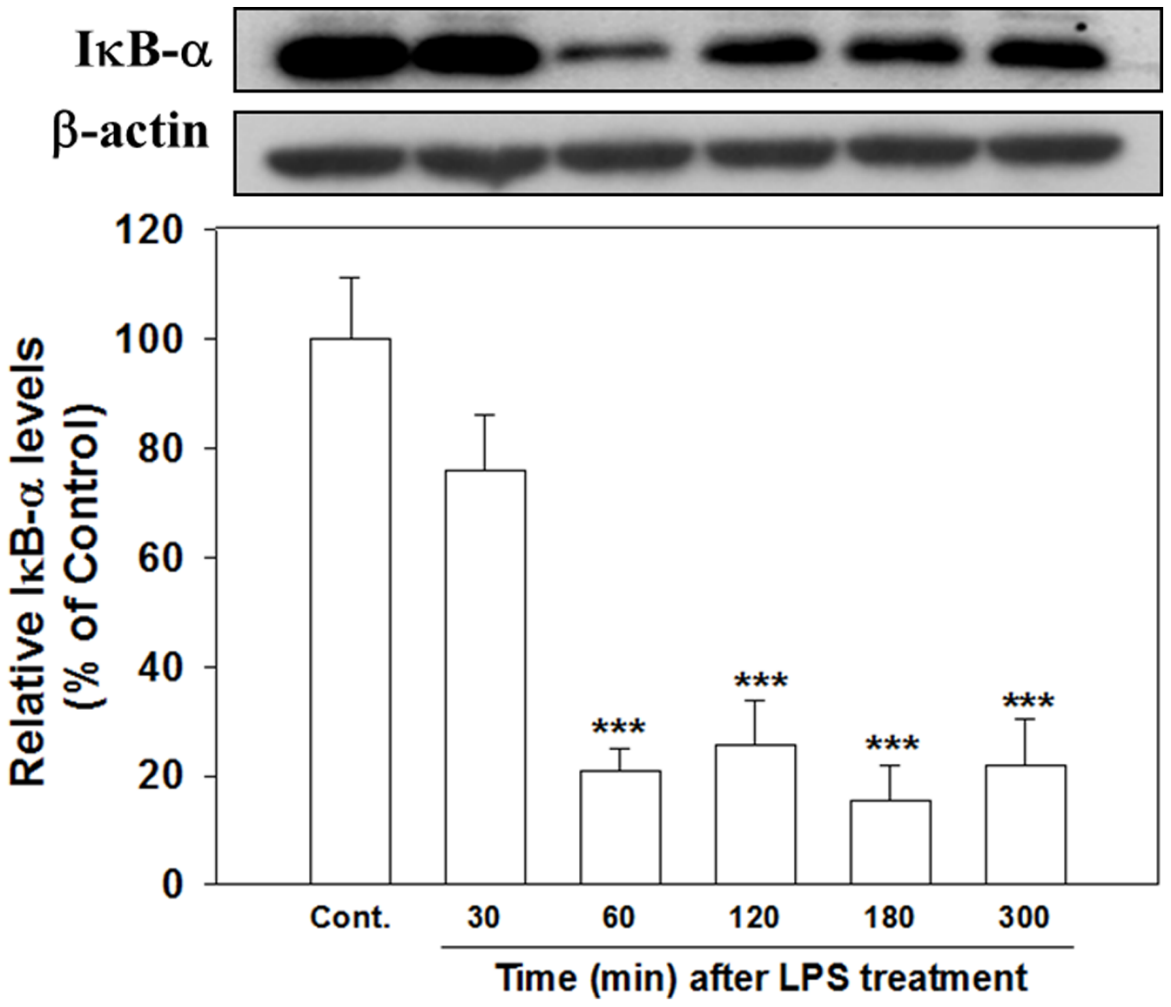
Time course of LPS-induced IκB-α degradation. Cells were treated with LPS (100 ng/ml) for various times and harvested to conduct western blot analyses of IκB-α degradation. LPS treatment suppressed IκB-α degradation as early as 60 min and was maintained at least until 300 min. The data are represented as the mean ± SEM based on 3–5 independent experiments. *** *p*<.001 versus Cont.

NF-κB is a nuclear activator instrumental in initiating immune responses at the nuclear level. In the resting state, it exists as a heterodimer of p65 and p50 bound in the cytoplasm to the cytosolic protein IκB-α, and NF-κB is dissociated upon stimulation before translocation. We explored how Vit. C cotreatment affected the LPS-induced stimulation of this vital nuclear activator from 2 perspectives: IκB-α dissociation/degradation and p65 nuclear translocation. IκB-α exhibited significant degradation at 60 min (Fig. 10; *p*<.001) after LPS treatment and these levels were maintained up to 300 min (Fig. 10; *p*<.001).

**Figure 11:**
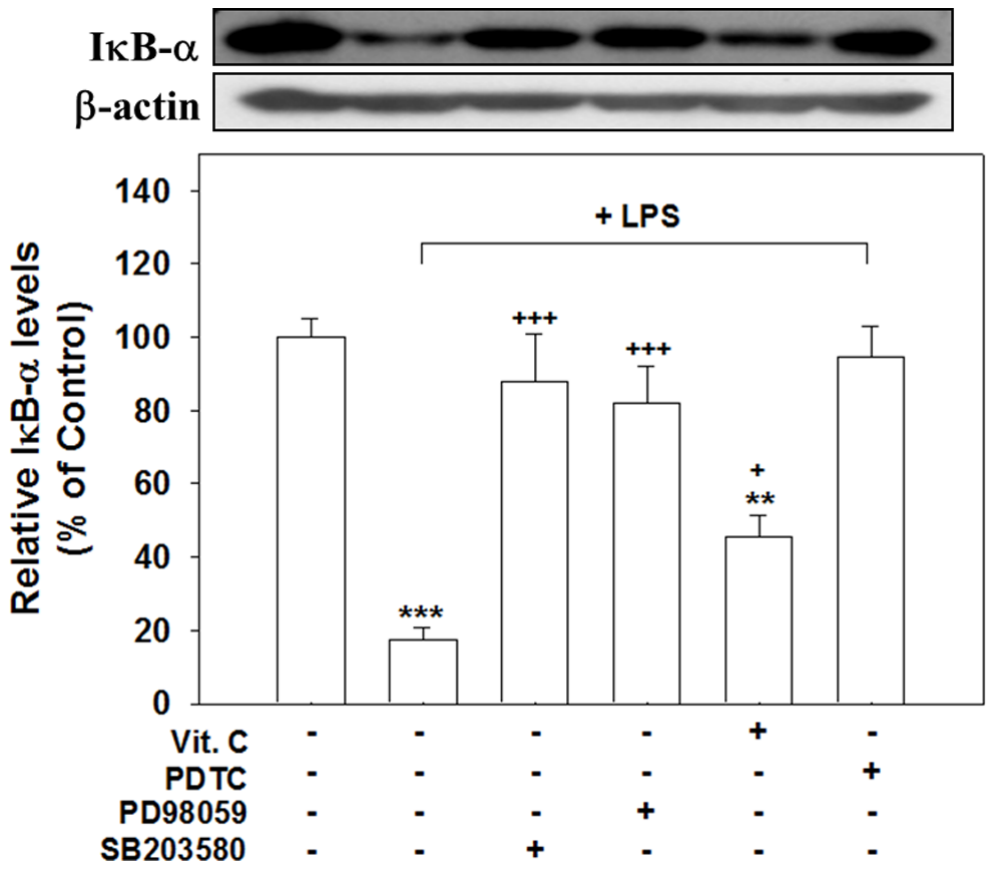
MAPK inhibitors, Vit. C, and PDTC inhibited the LPS induced degradation of IκB-α. Cultured cells were treated for 60(control), LPS alone (100 ng/ml), or simultaneous LPS with SB203580 (20 µM), PD98059 (20 µM), Vit. C (10 mM), or the NF-κB inhibitor PDTC (50 µM) and then harvested to conduct western blot analyses of the IκB-α degradation. The LPS-induced suppression of IκB-α degradation was significantly inhibited by cotreating all inhibitors and Vit. C, which exerted the least amount inhibition. The data are expressed as the mean ± SEM based on 4–5 experiments. ** *p*<.01 and *** *p*<.001 versus Cont.; ^+^ *p*<.05, ^+++^ *p*<.001 versus LPS alone.

Figure 11 shows that the degradation of IκB-α after 60 min of LPS treatment was significantly reversed by treatment with SB203580 (*p*<.001), PD98059 (*p*<.001), and PDTC (an NF-κB inhibitor; *p*<.001). Cotreatment with Vit. C also significantly attenuated the amount of LPS induced IκB-α degradation (*p*<.05), albeit not to the same degrees as the inhibitors.

**Figure 12:**
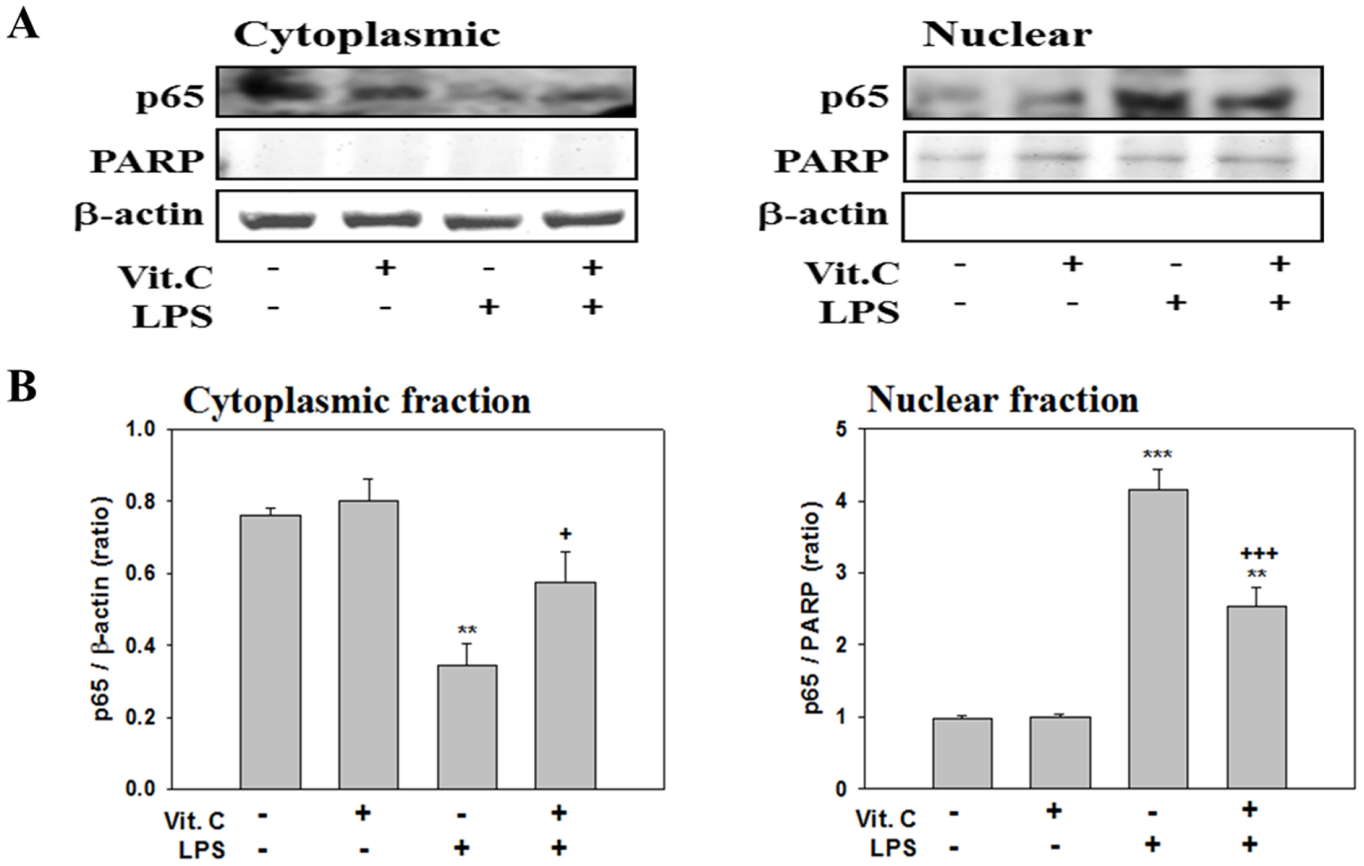
Vitamin C attenuated LPS-induced NF-κB translocation. (A) Gel images of the western blot analyses of the p65 protein extracted in cytoplasmic and nuclear fractions from the control cultures (no treatment) or cultures treated with Vit. C (10 mM) alone, LPS (100 ng/ml) alone, or combined Vit. C-LPS for 60 min. PARP (a nuclear loading control) and β-actin (a cytoplasmic loading control) were used as internal controls to ensure equal protein loading. (B) Quantitative comparison of the relative p65 levels in cytoplasmic and nuclear fractions extracted from cultures that underwent various treatments. The data are represented as the mean ± SEM based on 3 experiments. ** *p*<.01 and *** *p*<.001 versus Cont.; ^+^ *p*<.05, ^+++^ *p*<.001 versus LPS alone.

The degradation of IκB-α releases p65 and other transcription factors from cellular complexes, causing such factors to be translocated to nuclei. We examined the nuclear translocation of p65 by using western blotting. LPS treatment significantly shifted the presence of p65 from the cytoplasmic (*p*<.01) to the nuclear fraction (*p*<.001). The LPS effect was significantly attenuated after cotreatment with Vit. C (Fig. 12; *p*<.05 and *p*<.001). Vit. C treatment alone exerted no effects. These results suggest that suppression of the LPS-induced nuclear translocation of NF-κB is a mechanism underlying the Vit. C-induced attenuation of neuroinflammation.

## Discussion

The activation of glial cells following infection of the central nervous system or neuronal injury causes neuroinflammation, which involves a series of biological responses such as the production of proinflammatory mediators and morphological changes among glia cells. Excessive or chronic glial activation may exacerbate neuronal damage; therefore, identifying mechanisms that can control neuroinflammation should provide opportunities to interrupt inflammatory processes and prevent deleterious consequences. In this study, we used LPS, the major component of the outer membrane of Gram-negative bacteria, as a stimulant in neuron/glia cocultures to form a neuroinflammation model. In this model, LPS significantly and time-dependently induced the production of proinflammatory mediators such as NO (reflected by iNOS protein levels and nitrite accumulation), IL-6 (a cytokine) and MIP-2 (a chemokine) in cortical neuron/glia cocultures. Vit. C reduced the LPS-induced production of NO and proinflammatory mediators and attenuated LPS-induced glial activation. Regarding whether Vit. C interrupts LPS-elicited intracellular signaling, we demonstrated that LPS induced the phosphorylation of p38 and ERK MAPK and translocation of NF-κB. These signaling events were suppressed by Vit. C.

Neuroinflammation is also implicated in various neuronal pathologies, such as traumatic brain injury [25] and septic encephalopathy [26], and is accompanied by the accumulation of oxidative stress and depletion of the endogenous antioxidant Vit. C [11], [27]–[29]. A clinical study indicated that the levels of Vit. C in plasma and CSF were significantly decreased among patients with septic encephalopathy [29]. Reduced Vit. C levels in CSF correlate with the severity of neurologic symptoms [29]. These findings underscore the importance of Vit. C in the neuroinflammation induced through various insults. In this study, LPS-induced neuroinflammation was manifested based on the levels of iNOS expression, nitrite accumulation, and IL-6 and MIP-2 release in neuron/glial cells.

Vit. C is present at millimolar concentrations in neurons (10 mM) and glia (1 mM) [16], indicating that it plays a vital role in the brain. The major route by which Vit. C enters brain cells is from plasma to CSF through a Vit. C transporter (e.g., sodium-dependent Vit. C transporter). Various studies have demonstrated the functions of Vit. C in the brain such as the regulation of neural maturation [30], [31] and neuromodulation [32]. In addition, Vit. C exerts valuable neuroprotective effects against various brain insults such as stroke [21], Alzheimer's disease [22], and methamphetamine-induced neurotoxicity [33]. Using an *in vitro* neuroinflammatory model involving LPS in cultured neuron/glial cells enabled inducing unequivocal neuroinflammation and bypassing any possible physiological synergistic or feedback interactions in vivo. In this study, LPS-induced iNOS expression, nitrite accumulation, and IL-6 and MIP-2 production were significantly attenuated by treatment with Vit. C. To exclude the possibility that Vit. C attenuated the LPS-induced release of inflammatory mediators because of Vit. C toxicity, both LDH and MTT reduction assays were employed. Our results confirmed that millimolar concentrations of Vit. C were not toxic to cultured neuron/glial cells. The concentrations of Vit. C used in culture studies are typically in the millimolar range (1–20 mM) [18], [34] and are similar to those of other antioxidants such as N-acetylcysteine (NAC; 1–10 mM) [35], [36]. It is unclear why millimolar concentrations of antioxidants are required in order to exert physiological or pharmacological effects in culture systems.

It has been proposed that the attenuation of glial activation could yield therapeutic benefits in alleviating neuroinflammation. Various studies have indicated that Vit. C exerts inhibitory effects on glial activation. We determined that the astrocytes displayed hypertrophic and microglia displayed amoeboid morphologies (both characteristic of activated glia) after LPS treatment. However, the morphological features of activated glia were significantly attenuated by Vit. C. We speculate that Vit. C significantly attenuates the production of LPS-induced inflammatory mediators by inhibiting the LPS-induced activation of glial cells.

Although Vit. C exerts antiinflammatory effects on LPS-induced neuroinflammation, its underlying signaling pathways remain unclear. Studies have demonstrated the involvement of MAPK signaling pathways in the production of inflammatory mediators in neurons and glial cells [37]–[39]. The three major MAPK subfamilies are p38, ERK, and JNK. A previous study indicated that p38, ERK, and JNK are activated in both glia and neurons following LPS treatment [40]. The p38 and ERK MAPKs are related to LPS signaling in glial cells, leading to iNOS expression and the production of NO and proinflammatory cytokines [40]–[42]. The current results indicate that the phosphorylation of p38 and ERK MAPKs occurs after LPS treatment. MAPK activity inhibitors (SB203580 for p38 MAPK and PD98059 for ERK MAPK) were used to assess the involvement of MAPK pathways in the LPS-induced expression of iNOS and release of inflammatory mediators. Both MAPK inhibitors diminished the amount of LPS-induced iNOS expression, nitrite accumulation, and IL-6 and MIP-2 production, indicating that p38 and ERK MAPK are involved in the LPS-induced production of inflammatory mediators.

LPS can activate glia through Toll-like receptors, triggering downstream signaling, such as MAPK and NF-κB translocation, and resulting in the regulation of inflammatory responses [8], [9], [43]. LPS-activated MAPKs, such as p38 and ERK, are essential signaling cascades responsible for the increased production of inflammatory mediators in glial cells. We determined that Vit. C decreases the phosphorylation of p38 and ERK MAPK as a result of LPS. Therefore, the Vit. C-induced suppression of the production of inflammatory mediators may occur through the p38 and ERK pathways. In addition, NF-κB is a pleiotropic regulator of various genes involved in the production of inflammatory mediators. The transcription factor NF-κB is present in cytosol in an inactivate state in which it is bound to IκB protein. Degradation of IκB proteins activates NF-κB and its subsequent nuclear translocation. Studies have demonstrated that the activation of NF-κB in LPS-activated glial cells following the degradation of IκB-α increases the expression of downstream genes such as iNOS and IL-6 [44], [45]. In this study, Vit. C could inhibit the LPS-induced degradation of IκB-α and nuclear translocation of NF-κB p65. Our results are consistent with those of previous studies, which have reported that Vit. C can alter redox-sensitive signaling pathways to reduce inflammation [46]–[48].

In summation, these results suggest that Vit. C significantly attenuated the LPS-induced production of inflammatory mediators by inhibiting p38 and ERK MAPK signaling and the translocation of NF-κB in neuron/glia cocultures. These findings are notable because fruits and vegetables provide low-cost sources of Vit. C. Consuming a diet containing Vit. C might be an innovative approach to ameliorating neuroinflammation or symptoms resulting from acute insults to the central nervous system or chronic neurodegenerative diseases.

## References

[1] Wen LL, Chiu CT, Huang YN, Chang CF, Wang JY (2007) Rapid glia expression and release of proinflammatory cytokines in experimental Klebsiella pneumoniae meningoencephalitis. Exp Neurol 205: 270–278. 17397834 10.1016/j.expneurol.2007.02.011

[2] Chiu CT, Wen LL, Pao HP, Wang JY (2011) Cortistatin is induced in brain tissue and exerts neuroprotection in a rat model of bacterial meningoencephalitis. J Infect Dis 204: 1563–1572. 21940421 10.1093/infdis/jir608

[3] Raza SS, Khan MM, Ahmad A, Ashafaq M, Islam F, et al (2013) Neuroprotective effect of naringenin is mediated through suppression of NF-kappaB signaling pathway in experimental stroke. Neuroscience 230: 157–171. 23103795 10.1016/j.neuroscience.2012.10.041

[4] Woodcock T, Morganti-Kossmann MC (2013) The role of markers of inflammation in traumatic brain injury. Front Neurol 4: 18. 23459929 10.3389/fneur.2013.00018 PMC3586682

[5] Sochocka M, Koutsouraki ES, Gasiorowski K, Leszek J (2013) Vascular Oxidative Stress and Mitochondrial Failure in the Pathobiology of Alzheimer's Disease: New Approach to Therapy. CNS Neurol Disord Drug Targets. 10.2174/18715273113129990072 23469836

[6] O'Callaghan JP, Sriram K, Miller DB (2008) Defining “neuroinflammation”. Ann N Y Acad Sci 1139: 318–330. 18991877 10.1196/annals.1432.032

[7] Wang JY, Shum AY, Hwang CP (1998) Ethanol modulates induction of nitric oxide synthase in glial cells by endotoxin. Life Sci 63: 1571–1583. 9808068 10.1016/s0024-3205(98)00424-x

[8] Lu X, Ma L, Ruan L, Kong Y, Mou H, et al (2010) Resveratrol differentially modulates inflammatory responses of microglia and astrocytes. J Neuroinflammation 7: 46. 20712904 10.1186/1742-2094-7-46 PMC2936301

[9] Leow-Dyke S, Allen C, Denes A, Nilsson O, Maysami S, et al (2012) Neuronal Toll-like receptor 4 signaling induces brain endothelial activation and neutrophil transmigration in vitro. J Neuroinflammation 9: 230. 23034047 10.1186/1742-2094-9-230 PMC3481358

[10] Zhang F, Liu J, Shi JS (2010) Anti-inflammatory activities of resveratrol in the brain: role of resveratrol in microglial activation. Eur J Pharmacol 636: 1–7. 20361959 10.1016/j.ejphar.2010.03.043

[11] Korcok J, Wu F, Tyml K, Hammond RR, Wilson JX (2002) Sepsis inhibits reduction of dehydroascorbic acid and accumulation of ascorbate in astroglial cultures: intracellular ascorbate depletion increases nitric oxide synthase induction and glutamate uptake inhibition. J Neurochem 81: 185–193. 12067232 10.1046/j.1471-4159.2002.00814.x

[12] Kinuta Y, Kikuchi H, Ishikawa M, Kimura M, Itokawa Y (1989) Lipid peroxidation in focal cerebral ischemia. J Neurosurg 71: 421–429. 2769392 10.3171/jns.1989.71.3.0421

[13] Christen S, Schaper M, Lykkesfeldt J, Siegenthaler C, Bifrare YD, et al (2001) Oxidative stress in brain during experimental bacterial meningitis: differential effects of alpha-phenyl-tert-butyl nitrone and N-acetylcysteine treatment. Free Radic Biol Med 31: 754–762. 11557313 10.1016/s0891-5849(01)00642-6

[14] Hornig D (1975) Distribution of ascorbic acid, metabolites and analogues in man and animals. Ann N Y Acad Sci 258: 103–118. 1106295 10.1111/j.1749-6632.1975.tb29271.x

[15] Harrison FE, May JM (2009) Vitamin C function in the brain: vital role of the ascorbate transporter SVCT2. Free Radic Biol Med 46: 719–730. 19162177 10.1016/j.freeradbiomed.2008.12.018 PMC2649700

[16] Rice ME (2000) Ascorbate regulation and its neuroprotective role in the brain. Trends Neurosci 23: 209–216. 10782126 10.1016/s0166-2236(99)01543-x

[17] Hediger MA (2002) New view at C. Nat Med. 8: 445–446. 10.1038/nm0502-445 11984580

[18] Bowie AG, O'Neill LA (2000) Vitamin C inhibits NF-kappa B activation by TNF via the activation of p38 mitogen-activated protein kinase. J Immunol 165: 7180–7188. 11120850 10.4049/jimmunol.165.12.7180

[19] Carcamo JM, Borquez-Ojeda O, Golde DW (2002) Vitamin C inhibits granulocyte macrophage-colony-stimulating factor-induced signaling pathways. Blood 99: 3205–3212. 11964284 10.1182/blood.v99.9.3205

[20] Padh H (1990) Cellular functions of ascorbic acid. Biochem Cell Biol 68: 1166–1173. 2268411 10.1139/o90-173

[21] Huang J, Agus DB, Winfree CJ, Kiss S, Mack WJ, et al (2001) Dehydroascorbic acid, a blood-brain barrier transportable form of vitamin C, mediates potent cerebroprotection in experimental stroke. Proc Natl Acad Sci U S A 98: 11720–11724. 11573006 10.1073/pnas.171325998 PMC58796

[22] Rosales-Corral S, Tan DX, Reiter RJ, Valdivia-Velazquez M, Martinez-Barboza G, et al (2003) Orally administered melatonin reduces oxidative stress and proinflammatory cytokines induced by amyloid-beta peptide in rat brain: a comparative, in vivo study versus vitamin C and E. J Pineal Res. 35: 80–84. 10.1034/j.1600-079x.2003.00057.x 12887649

[23] Huang YN, Wu CH, Lin TC, Wang JY (2009) Methamphetamine induces heme oxygenase-1 expression in cortical neurons and glia to prevent its toxicity. Toxicol Appl Pharmacol 240: 315–326. 19576919 10.1016/j.taap.2009.06.021

[24] Mosmann T (1983) Rapid colorimetric assay for cellular growth and survival: application to proliferation and cytotoxicity assays. J Immunol Methods 65: 55–63. 6606682 10.1016/0022-1759(83)90303-4

[25] Bigler ED (2013) Neuroinflammation and the dynamic lesion in traumatic brain injury. Brain 136: 9–11. 23365089 10.1093/brain/aws342

[26] Semmler A, Hermann S, Mormann F, Weberpals M, Paxian SA, et al (2008) Sepsis causes neuroinflammation and concomitant decrease of cerebral metabolism. J Neuroinflammation 5: 38. 18793399 10.1186/1742-2094-5-38 PMC2553764

[27] Tyurin VA, Tyurina YY, Borisenko GG, Sokolova TV, Ritov VB, et al (2000) Oxidative stress following traumatic brain injury in rats: quantitation of biomarkers and detection of free radical intermediates. J Neurochem 75: 2178–2189. 11032908 10.1046/j.1471-4159.2000.0752178.x

[28] Bayir H, Kagan VE, Tyurina YY, Tyurin V, Ruppel RA, et al (2002) Assessment of antioxidant reserves and oxidative stress in cerebrospinal fluid after severe traumatic brain injury in infants and children. Pediatr Res 51: 571–578. 11978879 10.1203/00006450-200205000-00005

[29] Voigt K, Kontush A, Stuerenburg HJ, Muench-Harrach D, Hansen HC, et al (2002) Decreased plasma and cerebrospinal fluid ascorbate levels in patients with septic encephalopathy. Free Radic Res 36: 735–739. 12180123 10.1080/10715760290032557

[30] Lee JY, Chang MY, Park CH, Kim HY, Kim JH, et al (2003) Ascorbate-induced differentiation of embryonic cortical precursors into neurons and astrocytes. J Neurosci Res 73: 156–165. 12836158 10.1002/jnr.10647

[31] Shin DM, Ahn JI, Lee KH, Lee YS (2004) Ascorbic acid responsive genes during neuronal differentiation of embryonic stem cells. Neuroreport 15: 1959–1963. 15305145 10.1097/00001756-200408260-00025

[32] Rebec GV, Pierce RC (1994) A vitamin as neuromodulator: ascorbate release into the extracellular fluid of the brain regulates dopaminergic and glutamatergic transmission. Prog Neurobiol 43: 537–565. 7816935 10.1016/0301-0082(94)90052-3

[33] Huang YN, Wang JY, Lee CT, Lin CH, Lai CC (2012) L-ascorbate attenuates methamphetamine neurotoxicity through enhancing the induction of endogenous heme oxygenase-1. Toxicol Appl Pharmacol 265: 241–252. 23022510 10.1016/j.taap.2012.08.036

[34] Becker JC, Grosser N, Boknik P, Schroder H, Domschke W, et al (2003) Gastroprotection by vitamin C-a heme oxygenase-1-dependent mechanism? Biochem Biophys Res Commun 312: 507–512. 14637166 10.1016/j.bbrc.2003.10.146

[35] Ryu R, Shin Y, Choi JW, Min W, Ryu H, et al (2002) Depletion of intracellular glutathione mediates zinc-induced cell death in rat primary astrocytes. Exp Brain Res 143: 257–263. 11880902 10.1007/s00221-001-0991-7

[36] Lin CH, Kuo SC, Huang LJ, Gean PW (2006) Neuroprotective effect of N-acetylcysteine on neuronal apoptosis induced by a synthetic gingerdione compound: involvement of ERK and p38 phosphorylation. J Neurosci Res 84: 1485–1494. 16983658 10.1002/jnr.21047

[37] Gorina R, Font-Nieves M, Marquez-Kisinousky L, Santalucia T, Planas AM (2011) Astrocyte TLR4 activation induces a proinflammatory environment through the interplay between MyD88-dependent NFkappaB signaling, MAPK, and Jak1/Stat1 pathways. Glia 59: 242–255. 21125645 10.1002/glia.21094

[38] Zhao S, Zhang L, Lian G, Wang X, Zhang H, et al (2011) Sildenafil attenuates LPS-induced pro-inflammatory responses through down-regulation of intracellular ROS-related MAPK/NF-kappaB signaling pathways in N9 microglia. Int Immunopharmacol 11: 468–474. 21220057 10.1016/j.intimp.2010.12.017

[39] Kim WI, Ryu HJ, Kim JI, Seo CH, Lee BC, et al.. (2012) Differential nuclear factor-kappa B phosphorylation induced by lipopolysaccharide in the hippocampus of P2×7 receptor knockout mouse. Neurol Res. 10.1179/1743132812Y.0000000137 23540405

[40] Xie Z, Smith CJ, Van Eldik LJ (2004) Activated glia induce neuron death via MAP kinase signaling pathways involving JNK and p38. Glia 45: 170–179. 14730710 10.1002/glia.10314

[41] Ashwell JD (2006) The many paths to p38 mitogen-activated protein kinase activation in the immune system. Nat Rev Immunol 6: 532–540. 16799472 10.1038/nri1865

[42] Kim BW, Koppula S, Hong SS, Jeon SB, Kwon JH, et al (2013) Regulation of Microglia Activity by Glaucocalyxin-A: Attenuation of Lipopolysaccharide-Stimulated Neuroinflammation through NF-kappaB and p38 MAPK Signaling Pathways. PLoS One 8: e55792. 23393601 10.1371/journal.pone.0055792 PMC3564949

[43] Wang YP, Wu Y, Li LY, Zheng J, Liu RG, et al (2011) Aspirin-triggered lipoxin A4 attenuates LPS-induced pro-inflammatory responses by inhibiting activation of NF-kappaB and MAPKs in BV-2 microglial cells. J Neuroinflammation 8: 95. 21831303 10.1186/1742-2094-8-95 PMC3162900

[44] Giri S, Rattan R, Singh AK, Singh I (2004) The 15-deoxy-delta12,14-prostaglandin J2 inhibits the inflammatory response in primary rat astrocytes via down-regulating multiple steps in phosphatidylinositol 3-kinase-Akt-NF-kappaB-p300 pathway independent of peroxisome proliferator-activated receptor gamma. J Immunol 173: 5196–5208. 15470065 10.4049/jimmunol.173.8.5196

[45] Zhu C, Xiong Z, Chen X, Peng F, Hu X, et al (2012) Artemisinin attenuates lipopolysaccharide-stimulated proinflammatory responses by inhibiting NF-kappaB pathway in microglia cells. PLoS One 7: e35125. 22514713 10.1371/journal.pone.0035125 PMC3325975

[46] Son EW, Mo SJ, Rhee DK, Pyo S (2004) Vitamin C blocks TNF-alpha-induced NF-kappaB activation and ICAM-1 expression in human neuroblastoma cells. Arch Pharm Res 27: 1073–1079. 15554267

[47] Tan PH, Sagoo P, Chan C, Yates JB, Campbell J, et al (2005) Inhibition of NF-kappa B and oxidative pathways in human dendritic cells by antioxidative vitamins generates regulatory T cells. J Immunol 174: 7633–7644. 15944264 10.4049/jimmunol.174.12.7633

[48] Wilson JX (2009) Mechanism of action of vitamin C in sepsis: ascorbate modulates redox signaling in endothelium. Biofactors 35: 5–13. 19319840 10.1002/biof.7 PMC2767105

